# Supplementary material for: Investigation of MicroRNA Biomarkers in Equine Distal Interphalangeal Joint Osteoarthritis
Source: Int J Mol Sci. 2022 Dec 8;23(24):15526. doi: 10.3390/ijms232415526 (PMC9779011; doi:10.3390/ijms232415526)
Supplement: Supplementary file 1 [file ijms-23-15526-s001.zip › Supplementary data.pdf]

Supplementary data

Table S1. Primers for quantitative real-time polymerase chain reaction (RT-qPCR). Protein coding primers were exon spanning.

Gene	Primer sequence 5’-3’
**eca-miR-16**	UAGCAGCACGUAAAUAUUGGCG
**eca-miR-92a**	UAUUGCACUUGUCCCGGCCUGU
**eca-miR-27b**	UUCACAGUGGCUAAGUUCUGC
**U6 snRNA**	GeneGlobe ID YP02119464
**eca-ACAN**	F- CAACAACAATGCCCAAGACTAC<br>R- AGTTCTCAAATTGCAAGGAGTG
**eca-ADAMTS-4**	F-GCCTTTGGGGAGACGCTGCTACTA<br>R- GATGTGAGCCCCAGGTCCCCCAGC
**eca-ADAMTS-5**	F-AACTGGGGGTCCTGGGGGTCCTGG<br>R-CATTTCTTGCCTCACACTGCTCAT
**eca-COL1A2**	F-GCACATGCCGTGACTTGAGA<br>R-CATCCATAGTGCATCCTTGATTAGG
**eca-COL2A1**	F-TCAAGTCCCTCAACAACCAGATC<br>R-GTCAATCCAGTAGTCTCCGCTCTT
**eca-COL3A1**	F-ACGCAAGGCCGTGAGACTA<br>R-TGATCAGGACCACCAACATCA
**eca-COMP**	F- GGTGCGGCTGCTATGGAA<br>R- CCAGCTCAGGGCCCTCAT
**eca-GAPDH**	F-GCATCGTGGAGGGACTCA<br>R-GCCACATCTTCCCAGAGG
**eca-MMP-13**	F-GTCCCTGATGTGGGTGAATAC<br>R-ACATCAGACCAAACTTTGAAGG
**eca-RUNX2**	F-CTGGGCCATGTGTATGATTTGT<br>R- TTTTGACCTGATATAGAGTGCATGGT
**eca-SOX9**	F- CTTTGGTTTGTGTTCGTGTTTTGT<br>R-AGAGAAAGAAAAAGGGAAAGGTAAGTTT

Table S2 (excel file attached)

Data from the small RNA-sequencing showing all miRNAs detected in the equine synovial fluid samples.

Table S3. miRNAs significantly differentially expressed (FDR < 0.1) between mild and severe OA synovial fluid samples.

**miRNA gene name**	**LogFC**	**P value**	**FDR**	**Mean RPM mild OA synovial fluid samples**	**Mean RPM severe OA synovial fluid samples**
eca-miR-92a	-0.92	0.0052	0.0470	37744	28479
eca-miR-16	-0.92	0.0061	0.0470	27879	24882
eca-miR-25	-1.11	0.0052	0.0470	33356	28754

MicroRNA (miRNA), equine callabalus (eca), Log fold change, (LogFC), false discovery rate (FDR), reads per million (RPM) and osteoarthritis (OA).

Table S4. Results from Normfinder showing the stability value, standard error and mean RPM of miR-27b in the synovial fluid samples.

miRNA gene name	Stability value	Standard error	Mean RPM in synovial fluid samples
eca-miR-27b	0.346	0.051	10046

Table S5. Number of synovial fluid samples needed in the mild OA and severe OA group to obtain a power of 0.95 at a significance level of 0.05 for each miRNA.

miRNA	Number of samples needed for each group
miR-92a	36
miR-16	417
miR-25	2149
